# Impact of Adipose Tissue Depot Harvesting Site on the Multilineage Induction Capacity of Male Rat Adipose-Derived Mesenchymal Stem Cells: An In Vitro Study

**DOI:** 10.3390/ijms24087513

**Published:** 2023-04-19

**Authors:** Hussein M. El-Husseiny, Masahiro Kaneda, Eman A. Mady, Tadashi Yoshida, Ahmed S. Doghish, Ryou Tanaka

**Affiliations:** 1 Laboratory of Veterinary Surgery, Department of Veterinary Medicine, Faculty of Agriculture, Tokyo University of Agriculture and Technology, 3-5-8 Saiwai Cho, Fuchu-shi 183-8509, Tokyo, Japan; 2 Department of Surgery, Anesthesiology, and Radiology, Faculty of Veterinary Medicine, Benha University, Moshtohor, Toukh 13736, Elqaliobiya, Egypt; 3 Laboratory of Veterinary Anatomy, Division of Animal Life Sciences, Faculty of Agriculture, Tokyo University of Agriculture and Technology, 3-5-8 Saiwai Cho, Fuchu-shi 183-8509, Tokyo, Japan; 4 Department of Animal Hygiene, Behavior, and Management, Faculty of Veterinary Medicine, Benha University, Moshtohor, Toukh 13736, Elqaliobiya, Egypt; 5 Department of Applied Biological Science, Tokyo University of Agriculture and Technology, Tokyo 183-8509, Japan; 6 Department of Biochemistry, Faculty of Pharmacy, Badr University in Cairo (BUC), Badr City 11829, Cairo, Egypt; 7 Department of Biochemistry, Faculty of Pharmacy (Boys), Al-Azhar University, Nasr City 11651, Cairo, Egypt

**Keywords:** adipose stem cells, AdMSCs, differentiation, fat depots, harvesting site, male rats

## Abstract

Recently, substantial attention has been paid toward adipose-derived mesenchymal stem cells (AdMSCs) as a potential therapy in tissue engineering and regenerative medicine applications. Rat AdMSCs (r-AdMSCs) are frequently utilized. However, the influence of the adipose depot site on the multilineage differentiation potential of the r-AdMSCs is still ambiguous. Hence, the main objective of this study was to explore the influence of the adipose tissue harvesting location on the ability of r-AdMSCs to express the stem-cell-related markers and pluripotency genes, as well as their differentiation capacity, for the first time. Herein, we have isolated r-AdMSCs from the inguinal, epididymal, peri-renal, and back subcutaneous fats. Cells were compared in terms of their phenotype, immunophenotype, and expression of pluripotency genes using RT-PCR. Additionally, we investigated their potential for multilineage (adipogenic, osteogenic, and chondrogenic) induction using special stains confirmed by the expression of the related genes using RT-qPCR. All cells could positively express stem cell marker CD 90 and CD 105 with no significant in-between differences. However, they did not express the hematopoietic markers as CD 34 and CD 45. All cells could be induced successfully. However, epididymal and inguinal cells presented the highest capacity for adipogenic and osteogenic differentiation (21.36-fold and 11.63-fold for OPN, 29.69-fold and 26.68-fold for BMP2, and 37.67-fold and 22.35-fold for BSP, respectively, in epididymal and inguinal cells (*p* < 0.0001)). On the contrary, the subcutaneous cells exhibited a superior potential for chondrogenesis over the other sites (8.9-fold for CHM1 and 5.93-fold for ACAN, (*p* < 0.0001)). In conclusion, the adipose tissue harvesting site could influence the differentiation capacity of the isolated AdMSCs. To enhance the results of their employment in various regenerative cell-based therapies, it is thus vital to take the collection site selection into consideration.

## 1. Introduction

Recent breakthroughs in biomedicine and tissue regeneration have significantly broadened the scope of healthcare [1,2,3]. Over the years, a wide range of therapeutic modalities and materials have been employed [4,5,6,7,8]. Mesenchymal stem cells (MSCs) are among the highly investigated cell sources for regenerative medicine and tissue engineering applications [9,10]. They can be obtained from diverse adult tissues originating from the mesodermal layer of embryos including but not limited to adipose tissue, bone marrow, umbilical cord blood, dental pulp, periosteum, muscle tissues, and periodontal ligament [11,12,13]. MSCs present various unique properties such as plastic adherent growth, high proliferation capacity, outstanding ability to differentiate into various cell lineages, and the expression of multiple cell surface markers [14]. Adipose tissue is a potent and facile source of adult multipotent stem cells used for regenerative medicine and tissue engineering purposes. Hence, adipose-derived MSCs (AdMSCs) are characterized by their outstanding plastic adherence proliferation [15] and their unique multilineage differentiation into various tissues such as adipose, osseous, cartilaginous, tendonous, cardiac, skeletal muscle, smooth muscle, and neural and connective tissues [16,17,18,19,20,21]. These exceptional properties make AdMSCs highly valuable for tissue engineering purposes [22]. The anatomical site for the harvesting of AdMSCs is very important because it may affect the entire characteristics of the inner cells and improve the differentiation potential of the harvested cells [23,24]. Moreover, despite the diversity in stem cell sources, it is difficult to attain the desired proliferation and differentiation capacities following implantation. Hence, it is crucial to choose an optimal adipose tissue source among various depots for optimal cell-based therapeutic outcomes. However, this process has numerous challenges [25]. Various fat depots can contribute to function and disease in variable ways, and this variance is attributed to regional variations in the types of cells and the intrinsic characteristics of the adipocyte progenitors [25]. Furthermore, many previous investigations have declared that the MSCs obtained from different sources are heterogenous and the donor and the source affect their differentiation potential [26,27,28]. Animal models are necessary for the study of adipose-derived stem cells and their applications. Among them are rats, which are a good source of MSCs. Rat adipose-derived MSCs (r-AdMSCs) are extensively employed in tissue engineering investigations [29,30]. Moreover, it has been reported that, unlike most other animal models, rats possess a relatively large amount of visceral fat that can be harvested to isolate MSCs that can be further directed to differentiate in diverse cell lineages based on the target application [29]. However, significant variations between rat MSCs have been found among r-AdMSCs. Moreover, knowledge about the influence of the different anatomical sites utilized for the isolation of r-AdMSCs in male rats on their differentiation is still limited. Moreover, previous research endeavors utilized the r-AdMSCs obtained from male rats regardless of the effect of the fat depot collection site. Thus, in the present article, we provide a comprehensive comparison of the r-AdMSCs isolated from different adipose sites in male rats and how this can affect the multilineage differentiation potential of the collected stem cells and consequently their application in tissue engineering and regenerative medicine.

## 2. Results

### 2.1. Morphologic and Immunophenotypic Characterization of Different Sites’ r-AdMSCs

**Figure 1:**
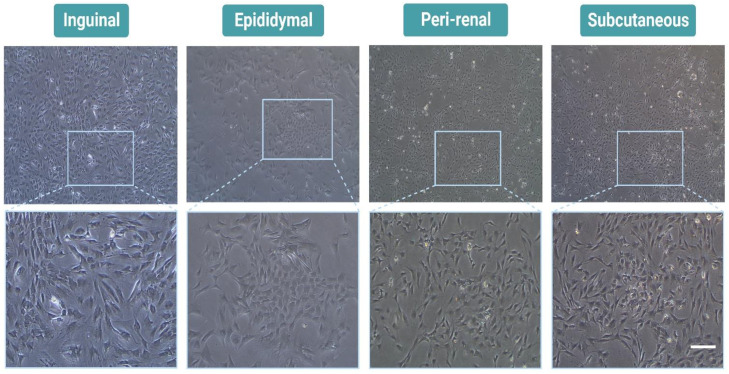
Representative images of the morphology of the r-AdMSCs harvested from different adipose tissue depots at the third passage (P3) and ~80% confluence. Scale bar: 200 µm.

**Figure 2:**
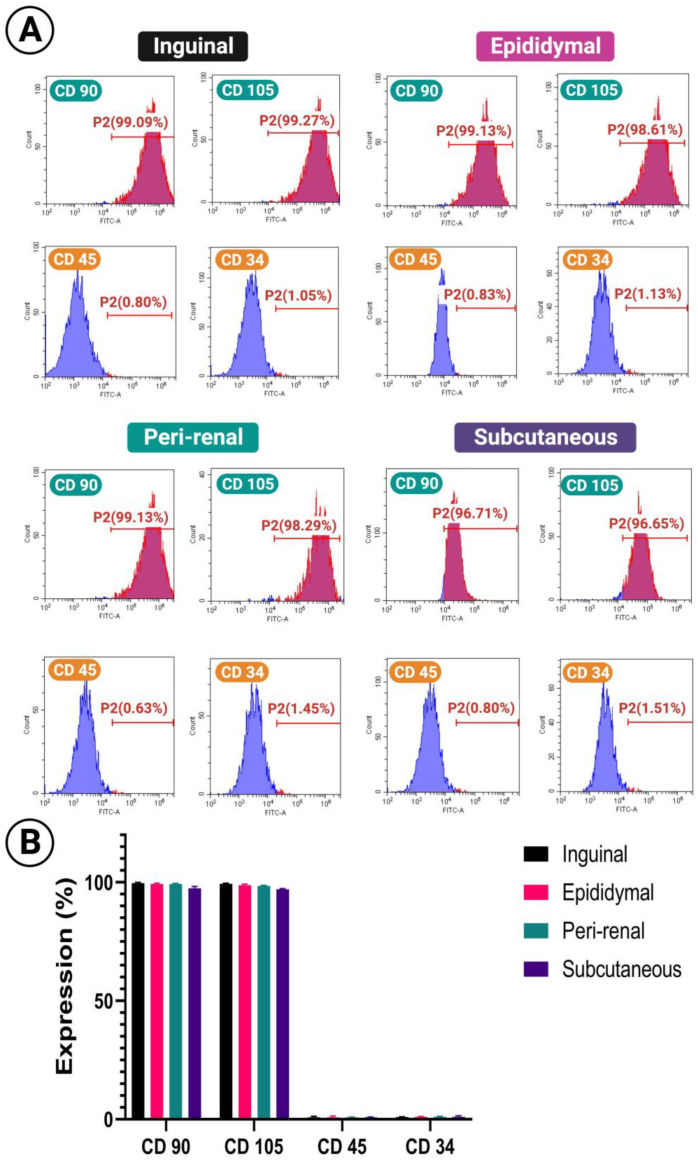
Expression of different cell surface markers in the cultured third-passage cells isolated from different adipose tissue harvesting sites in male SD rats. (**A**) Representative histograms for the expression of various surface markers: CD 90 and CD 105 with positive expression, and CD 45 and CD34 with negative expression. (**B**) Values of the expression % of each marker. Data were analyzed via one-way analysis of variance (ANOVA) followed by Tukey’s post hoc test and presented as mean ± SEM. No significance was detected.

A microscopic evaluation of the morphology of r-AdMSCs harvested from inguinal, epididymal, peri-renal, and subcutaneous adipose tissues is presented in Figure 1. The r-AdMSCs isolated from adipose tissues harvested from various sites had a unique plastic plate adhesion property, which was easily visualized using an inverted microscope, as illustrated in Figure 1. Most of the cells exhibited a fibroblast-like morphology. However, they also presented heterogeneous shapes of small round morphology in inguinal and some epididymal cells and spindle-shaped cells in peri-renal and subcutaneous cells. Most of the epididymal cells had the morphology of long fibroblast-like cells and presented a unique formation of cell clusters (Figure 1). The immune phenotypic characterization of the isolated cells from diverse locations is shown in Figure 2. All r-AdMSCs derived from diverse sites positively expressed the MSC marker (CD 90) and the endoglin marker (CD 105) (Figure 2A) with no significant differences observed between the different sites (Figure 2B). On the other hand, none of the cells attained from diverse locations expressed the hematopoietic markers (CD 34 and CD 45) (Figure 2).

### 2.2. Potential to Express the Pluripotency Genes via Different Sites’ r-AdMSCs

**Figure 3:**
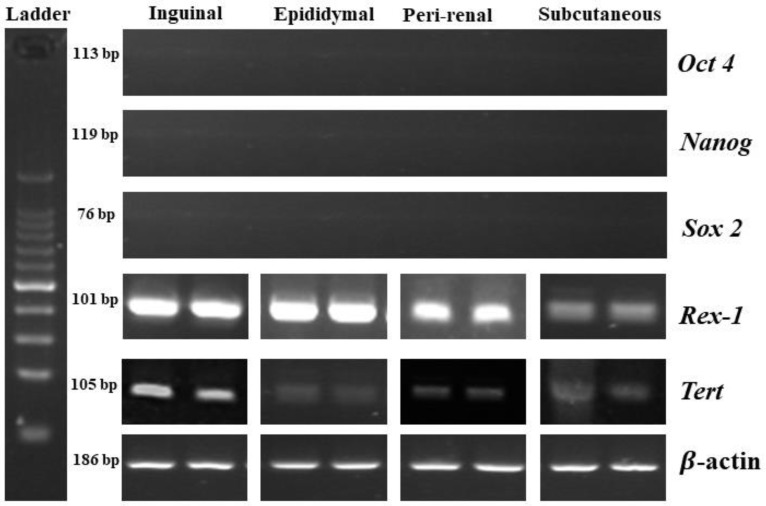
Expression of the pluripotency genes measured via RT-PCR in different cell populations harvested from diverse fat depot sites in male SD rats.

The capacity of different sites’ r-AdMSCs to express the pluripotency genes (*Oct 4*, *Nanog*, *Sox 2*, *Rex-1*, and *Tert*) is presented in Figure 3. *Oct 4*, *Nanog*, and *Sox 2* were not expressed by any of the cell populations. On the contrary, a positive expression of *Rex-1* and *Tert* was observed in diverse cells from various sites (Figure 3).

### 2.3. Qualitative and Quantitative Assessment of the Multilineage Differentiation Capacity of Different Sites’ r-AdMSCs

Cultured cells at the third passage from different adipose tissue depots were induced under different induction environments to differentiate into various cell lineages (adipogenic, osteogenic, and chondrogenic). The qualitative and quantitative evaluations of the multilineage differentiation capacity of the stem cells yielded from various anatomical sites (inguinal, epididymal, peri-renal, and subcutaneous) in male SD rats were as follows:

#### 2.3.1. Adipogenic Differentiation Potential

**Figure 4:**
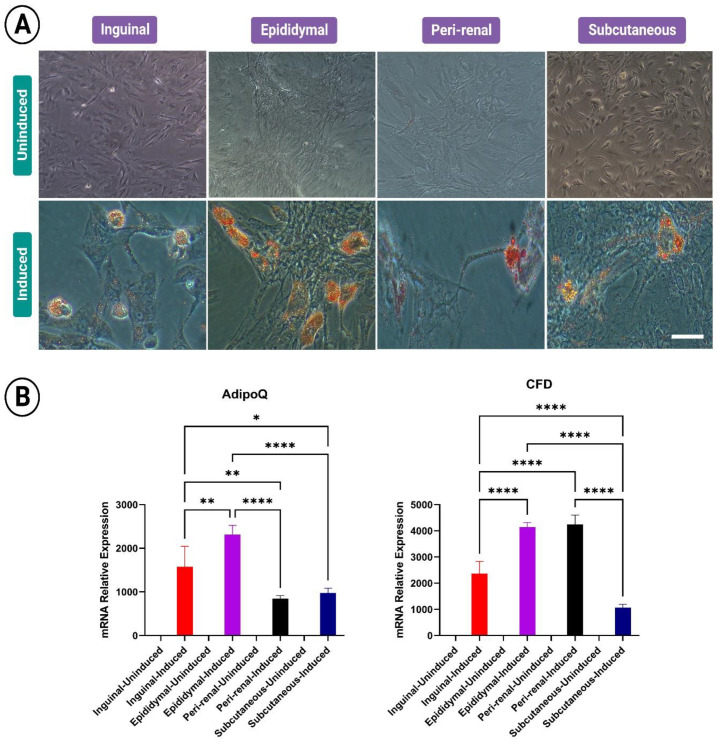
Adipogenic induction of the r-AdMSCs isolated from diverse harvesting sites. (**A**) Representative light microscopic images of the third-passage cultured induced and uninduced cells for qualitative assessment of the adipogenic differentiation. (**B**) Quantitative assessment of the adipogenic induction genes: Adiponectin (AdipoQ) and complement factor D (CFD). Scale bar: 200 µm. Data were analyzed via one-way analysis of variance (ANOVA) followed by Tukey’s post hoc test and were presented as mean ± SEM. * *p* < 0.05, ** *p* < 0.01, and **** *p* < 0.0001. The presented data are representative of four independent experiments.

Qualitative oil red O staining findings of the adipogenic differentiation of different cell depots are shown in Figure 4A. There was an obvious adipogenic induction of different depots with variable degrees. The adipocyte formation was indicated by the oil red O stain as round red droplets. The intensity of adipogenesis was the highest in the epididymal site followed by the inguinal site compared with the other two sites (peri-renal and subcutaneous). Moreover, adipocytes were not detected in the uninduced cells from different sites (Figure 4A). The qualitative assessment of the expression level of the adipocytic genes among the four sites of r-AdMSCs is shown in Figure 4B. There was a significant elevation in the expression of AdipoQ in the epididymal cells compared with the other cells, followed by the inguinal site, which was markedly higher than the subcutaneous and peri-renal sites. On the other hand, both the epididymal and the peri-renal sites presented a significantly higher expression of CFD than the inguinal and subcutaneous sites. The subcutaneous site presented the lowest expression of both adipogenic genes (AdipoQ and CFD) (Figure 4B). These results suggest that the best adipogenic induction was detected in the epididymal site (Figure 4).

#### 2.3.2. Osteogenic Differentiation Potential

**Figure 5:**
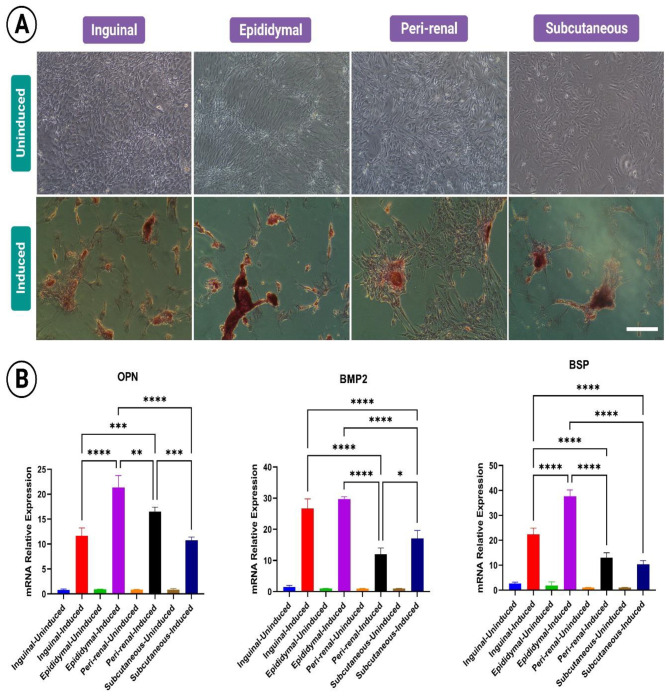
Osteonic induction of the r-AdMSCs isolated from diverse harvesting sites. (**A**) Representative light microscopic images of the third-passage cultured induced and uninduced cells for qualitative assessment of the osteogenic differentiation. (**B**) Quantitative assessment of the osteogenic induction genes Osteopontin (OPN), bone morphogenetic protein (BMP2), and bone sialoprotein (BSP). Scale bar: 200 µm. Data were analyzed via one-way analysis of variance (ANOVA) followed by Tukey’s post hoc test and were presented as mean ± SEM. * *p* < 0.05, ** *p* < 0.01, *** *p* < 0.001, and **** *p* < 0.0001. The presented data are representative of four independent experiments.

The osteogenic differentiation capacity of r-AdMSCs gained from variable locations was qualitatively assessed using alizarin red S stain as shown in Figure 5A. Over the time of the osteogenic induction, the cells quickly proliferated, clumped, and formed dense colonies with increased mineralization of their matrices throughout osteo-induction. The r-AdMSCs from the epididymal and inguinal sites presented the best osteo-induction reflected by the ALZ stain, followed by the peri-renal and the subcutaneous sites. We did not observe any evidence of osteo-induction in the uninduced control cells (Figure 5A).

The quantification values of the expression of osteogenic genes of the cultured cells from variable sites are presented in Figure 5B. The induced cells from different sites had a variable degree in the expression of the osteogenic genes. Epididymal cells showed the highest degree of OPN, BMP2, and BSP expression. There was a significant elevation (*p* < 0.05) in the expression of OPN and BSP in the epididymal cells compared to the other cells. No significant difference was recorded in the expression of BMP2 between the epididymal and inguinal cells, where cells from these two sites had a marked increase in the expression of BMP2 compared to the peri-renal and the subcutaneous sites. The inguinal cells presented a significantly increased expression of OPN, BMP2, and BSP compared to those of the peri-renal and subcutaneous sites. These results indicate that both the epididymal and inguinal sites had the best osteo-induction potentials (Figure 5).

#### 2.3.3. Chondrogenic Differentiation Potential

**Figure 6:**
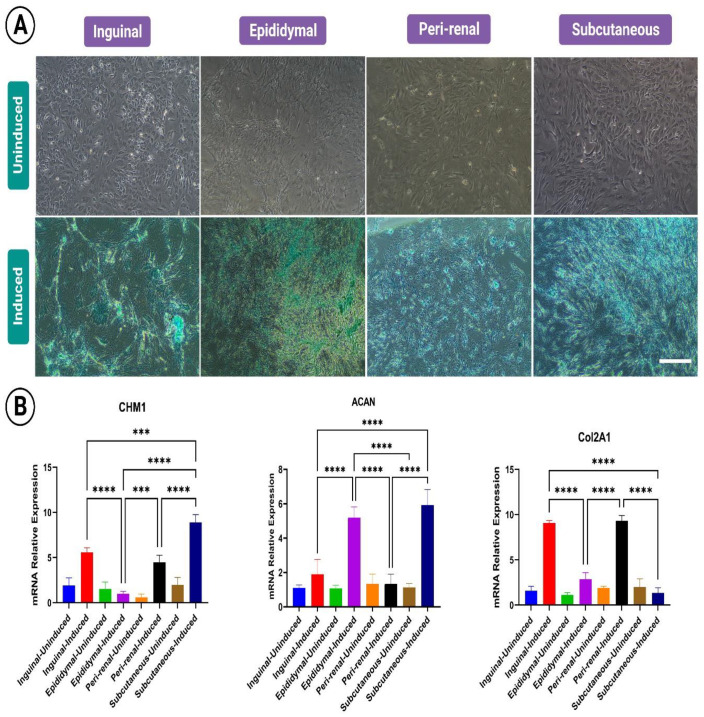
Chondrogenic induction of the r-AdMSCs isolated from diverse harvesting sites. (**A**) Representative light microscopic images of the third-passage cultured induced and uninduced cells for qualitative assessment of the chondrogenic differentiation. (**B**) Quantitative assessment of the chondrogenic induction genes Chondromodulin-1 (CHM1), Aggrecan (ACAN), and Collagen type 2 (Col2A1). Scale bar: 200 µm. Data were analyzed via one-way analysis of variance (ANOVA) followed by Tukey’s post hoc test and presented as mean ± SEM. *** *p* < 0.001 and **** *p* < 0.0001. The presented data are representative of four independent experiments.

Figure 6 shows the different capacities of r-AdMSCs from diverse sites for chondro-induction. Qualitative evaluation of the alcian blue staining confirmed the ability of cells from different fat depots to be induced for differentiation in the chondrogenic lineage. The induced cells appeared to be round, with low cell density, and had cartilaginous matrix depositions. These alterations were observed in the uninduced control cells. The density of staining was more obvious in the subcutaneous and epididymal cells followed by the inguinal and peri-renal cells (Figure 6A). The quantification of the expression levels of the chondrogenic genes showed that the subcutaneous cells had a significantly higher (*p* < 0.05) expression of CHM1 than the other cells and ACAN than the inguinal and peri-renal sites. Moreover, the inguinal cells presented a marked overexpression of CHM1 compared to the epididymal or the peri-renal sites. On the contrary, the epididymal cells had a remarkably higher expression of ACAN than the inguinal or the peri-renal cells. Meanwhile, the expression of Col2A1 was significantly higher in the inguinal and the peri-renal cells than in the other sites (Figure 6B). These findings imply that the subcutaneous cells had the highest potential for chondrogenic lineage induction followed by the inguinal, the epididymal, and the peri-renal cells (Figure 6).

## 3. Discussion

Recently, considerable attention has been paid toward AdMSCs as a potent therapeutic modality in various applications of tissue engineering and regenerative medicine. In particular, r-AdMSCs are a potential cell source for utilization in diverse cell therapies. Hence, various research endeavors have been directed toward the elaboration of their growth, heterogeneity, and differentiation processes [31]. In the present study, we compared four different cell populations that were harvested and isolated from various anatomical locations in male SD rats. We investigated their immune phenotypic characteristics, their pluripotency, and their potential to be induced for differentiation into various cell lineages. To the best of our knowledge, this is the first study to report on the variations in the adipogenic, osteogenic, and chondrogenic differentiation potentials of r-AdMSCs harvested from diverse fat depot sites (inguinal, epididymal, peri-renal, and subcutaneous) in male SD rats. Moreover, we compared the subcutaneous and various visceral adipose tissues that were situated differently and they possessed divergent metabolism and proliferation rates [32]. The adipose tissue can be harvested to isolate r-AdMSCs from diverse locations distributed throughout the bodies of male rats. In the current study, we chose the inguinal, epididymal, peri-renal, and subcutaneous fat depot sites from male SD rats, which are not often investigated. We successfully harvested, isolated, and expanded the r-AdMSCs from all of these sites. All of the cultures were found to be homogenous and presented the characteristic fibroblast-like morphology. Moreover, their expansion in vitro was facile under the normal culture environment, as described previously, with no need for extra specific conditions [33]. In the present study, the capacity of the cells isolated from different sites was tested to express the MSC-specific surface marker CD 90, endoglin, and the hematopoietic surface markers CD 45 and CD 34. These specific markers are frequently employed to characterize MSCs in rodent models [34,35,36]. According to our findings, all third-passage cells isolated from different fat depot sites positively expressed CD 90 and CD 105 with no significant differences between them. On the contrary, the expression of CD 45 and CD 34 was negative for all of the cells except for fine expression in some instances that indicated minor contamination with hematopoietic cells. These results are in line with [34,35,37], which reported that AdMSCs could lose their potential to express hematopoietic surface markers with subsequent passages. Regarding the pluripotency genes (*Oct 4*, *Nanog*, *Sox 2*, *Rex-1*, and *Tert*), the issue of their expression by AdMSCs is still controversial [38]. In embryonic stem cells, the expression of *Oct 4*, *Nanog*, and *Sox 2* is crucial to regulate differentiation and self-renewal. The molecular basis for the growth and pluripotency of embryonic stem cells is well understood, but the findings are contradictory [39]. In this study, we found that third-passage cells from diverse sites did not express any of these three genes. These findings are in line with [30,40] and consistent with another study [39] on adult human MSCs that declared that only *Nanog* was expressed. On the contrary, Castella et al. [41] reported the expression of these three genes in r-AdMSCs. On the other hand, in our experiment, cells from diverse sites expressed *Rex-1* and *Tert*. *Rex-1* is an important stem cell marker that has a key role in the differentiation of embryonic stem cells [42]. Additionally, *Tert* is crucial for enhancing the growth of primary cells and boosting the efficacy of the reprogramming of the induced pluripotent stem cells [43]. As multipotent cells, MSCs exhibit an outstanding ability to be induced and differentiate into various cell lineages, such as adipogenic, osteogenic, and chondrogenic lineages. Our findings demonstrated that r-AdMSCs from various locations differentiated into adipocytes, osteocytes, and chondrocytes. This is in agreement with [29]. However, the differentiation capacity was different among them. Moreover, we found that the quantitative results of the RT-qPCR were consistent with the qualitative findings of cell-specific staining to assess the differentiation capabilities. The findings confirm that the cell harvesting site is an important factor that influences the multilineage differentiation capacity that is crucial for the translation of stem cells into various tissue engineering applications. Moreover, we noticed that this variation in the differentiation potential of r-AdMSCs was not related to the similarities in the morphological or the immunophenotypic characteristics. This was attributed to the heterogeneity of the adipose tissue, which contains adipocytes and stromal vascular fraction (SVF) cells. Both adipocytes and SVF cells present an endocrine function and secrete cytokines with various biological effects [44]. Thus, each fat depot has a different profile of adipokines and interleukin secretion. An assessment of the fat depots from various sites showed diverse location-related characteristics of the adipose tissue and the interplay of paracrine between the adipose tissue and the surrounding tissues [45]. Investigations into human AdMSCs revealed that the harvesting site was an important factor that affected the properties of isolated cells. Moreover, every adipose depot has a definite embryonic origin and expresses a unique HOX code. This transcription factor is vital during the embryonic development of cells and is responsible for guiding cell differentiation toward the corresponding lineage, which is crucial for preserving the ideal functionality of diverse body systems. In addition, the HOX code is essential for the regulation of mature cells in adults and for defining the fat cells’ stem cell positional uniqueness [46,47]. In another work [46], it was revealed that knee, thigh, and breast fat depot sites possess diverse embryonic origins. Likewise, it has been found that knee and chin adipose tissues have dissimilar embryonic sources and could express divergent HOX codes [47]. To confirm the safety and enhance the outcomes of adipose tissue autografting, it is necessary to match the HOX code and the embryonic origin of the donor fat depot harvesting site and the receiving tissue [44]. All of these findings show that the AdMSCs isolated from fat depots from diverse harvesting sites have dissimilar embryonic origins, which affect their differentiation potentials. Notably, the existence of lineage “imprinting” in several stromal cell compartments that affects the differentiation potential of MSCs may potentially account for the variation in the differentiation potential between the examined harvesting locations, as previously demonstrated [48,49]. In the present study, r-AdMSCs isolated from epididymal, and inguinal harvesting sites exhibited the highest capacity of adipogenic differentiation indicated by oil red O staining and this was confirmed by their upregulation of AdipoQ and CFD expression with superiority toward the epididymal r-ADMSCs. These findings are in line with [50], whereby the authors found that the epididymal AdMSCs of mice started adipogenesis about 8 weeks earlier than the inguinal ones. This was attributed to the postnatal development of gonadal fat. However, the subcutaneous inguinal fat develops during the embryonic stages. Moreover, the de novo adipogenesis of the subcutaneous and visceral adipose tissues is still controversial [51]. Aaron et al. [52] declared that adipogenic progenitor cells had a higher abundance and proliferation in inguinal subcutaneous fat than epididymal adipose tissue harvested from mice fed on a high-fat diet. On the other hand, Pan et al. [53] declared that the higher potential of inguinal cells for adipogenic differentiation is related to their higher capacity in mitotic clonal expansion. Regarding the osteo-induction, the epididymal and inguinal cells had a superior potential for osteogenic differentiation followed by the peri-renal cells. This was confirmed via ALZ staining and the quantification of their ability to express the osteogenic-related genes (*OPN*, *BMP2*, and *BSP*). These results are consistent with those reported by [54,55]. Additionally, these findings are in line with [56], whereby the authors reported that visceral adipose tissues had higher abilities than subcutaneous ones to undergo osteogenic differentiation. This was attributed to variation in the histology and cell niches of the AdMSCs derived from adipose tissue sites. Moreover, the higher density of blood supply to visceral fat plays an important role in the elevated viability and stemness of their derived cells. Habib et al. [57] revealed that the adipose stem cells isolated from the epididymal fat presented potent osteogenic potential and could be a promising cell-based therapy to repair bone defects. These cells presented an elevated expression of the osteoblastic markers *RUNX-2*, *FOXO-1*, and *OC* and a decreased expression of the osteoclastic RANK gene. In another study, inguinal stem cells isolated from GFP mice exhibited outstanding osteogenic and chondrogenic differentiation [58]. This was attributed to the presence of the osteo-chondral precursor cells, similar to those of the human ADMSCs. The osteogenic differentiation capacity of the peri-renal r-ADMSCs was attributed to the blood and lymph supply in addition to the innervation of the peri-renal fat that is situated near to the kidneys [59]. Moreover, the elevated activity of renin-angiotensin in the peri-renal adipose tissue impacts the growth and the differentiation potential of the adipocytes [60,61]. Thus, the hormones are a factor that may direct the differentiation of adipose-derived stem cells. Wei K. et al. [62] attributed the osteogenic potential of the peri-renal stem cells to the complex relationship between bones and kidneys, the function of kidneys in the regulation of bone formation and metabolism, and the maintenance of calcium and phosphorus homeostasis, which is necessary for bone mineralization and development. On the other hand, alcian blue staining and the upregulated expression of the chondrogenic genes (*CHM1*, *ACAN*, and *Col2A1*) revealed that the subcutaneous, epididymal, and inguinal cells exhibited the highest capacity for chondrogenesis. These findings are in agreement with [63], whereby the authors declared that inguinal adipose tissue comprises heterogenic cell populations, which encompass osteogenic and chondrogenic progenitor cells with a high capacity for osteo-chondral induction. Likewise, Bo et al. [64] revealed that murine AdMSCs could effectively exhibit multilineage differentiation attributed to their content of diverse-lineage-dedicated progenitor cells such as pre-adipocytes, pre-osteoblasts, and other multipotent cells from additional sources such as pericytes, blood vessels, or a mixture of them.

## 4. Materials and Methods

### 4.1. Experimental Animals

Five healthy male SD rats of two months of age and 250–300 g in weight were used in the present study. We selected male rats to obtain epididymal adipose tissue in addition to inguinal, peri-renal, and back subcutaneous adipose tissues. Rats were housed in cages in a room at 20–25 °C and 40–60% humidity, with a 12 h light/dark cycle. Animals were fed plenty of dry food (Oriental Yeast Co., Ltd., Tokyo, Japan), which provided 359 kcal/100 g energy and 23.1 g/100 g protein, and water. Animals were euthanatized via an overdose of isoflurane according to [65]. All procedures were carried out following the Tokyo University of Agriculture and Technology’s Institutional Animal Care and Use Committee’s assessment and approval (approval no. R05-89).

### 4.2. Isolation and In Vitro Culture of Different Sites’ r-AdMSCs

**Figure 7:**
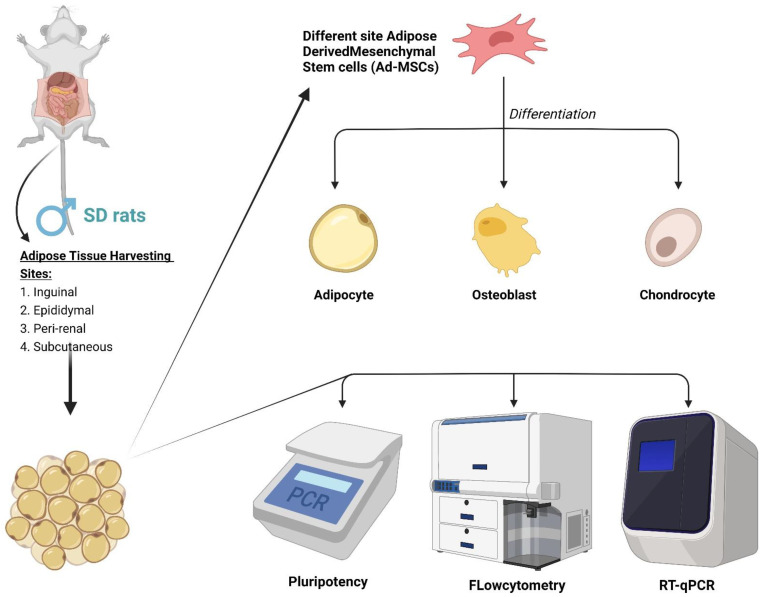
Schematic illustration of the present study design showing the isolation of the male Sprague Dawley (SD) rat adipose-derived mesenchymal stem cells (r-AdMSCs) from diverse fat depot harvesting sites followed by evaluation of the isolated cells using flowcytometry for immunophenotypic characterization of the isolated r-AdMSCs and PCR to qualitatively assess the expression of the pluripotent genes, and assessment of the differentiation potential of the isolated cells for adipogenic, osteogenic, and chondrogenic lineages’ induction both qualitatively, using specific stains, and quantitatively, using real-time qPCR.

Four different sites were selected to harvest the epididymal, inguinal, peri-renal, and back subcutaneous adipose tissue under strict sterile conditions (Figure 7). The obtained adipose tissue was thoroughly cleansed with PBS before being put in a sterile culture plate and diced using sterile scissors. After that, chopped adipose tissue was put into a shaking bath of Hank’s balanced salt solution (HBSS, 14175-095, 500 mL, Life Technologies Corporation, New York, NY, USA) with 0.1% (*w*/*v*) collagenase type 1 (1 mg/mL; Gibco by Life Technologies, Waltham, MA, USA) for 1 h at 37 °C. We added cold HBSS to counteract the effects of the collagenase. We attained a high-density cell pellet after centrifugation (800× *g* for 10 min). To eliminate the solid aggregates, 100 µm filters (BD Falcon, Bedford, MA, USA) were used. The pellet was resuspended, and the RBCs were lysed using a 1 mL RBCs lysis buffer (Promega, Mannheim, Germany). The cell pellet was then resuspended in Dulbecco’s Modified Eagle Medium (DMEM, 043-30085, Fujifilm Wako Pure Chemical Corporation, Osaka, Japan), which served as the foundation for the culture. This medium contained 4500 mg/L D-glucose, 4 mM L-glutamine, 110 mg/L sodium pyruvate, 10% fetal bovine serum (FBS, F2442-500ML, Sigma Aldrich, St. Louis, MI, USA), 1% non-essential amino acids, and 1% penicillin-streptomycin (Penicillin, Streptomycin, Amphotericin B suspension (×100), 161-23181, Fujifilm Wako Pure Chemical Corporation, Osaka, Japan) [55]. Unless otherwise noted, all compounds were purchased from Sigma-Aldrich (St. Louis, MO, USA). Following isolation, cells were cultured on 75 cm^2^ Nunclon culture dishes (Thermo Fisher Scientific, Waltham, MA, USA) at a density of 5 × 10^5^ cells/dish. In a humidified incubator, cells were grown under conditions of 37 °C, 95% humidity, and 5% CO_2_. The DMEM medium was switched out every three days until 80% confluency was reached. After separation using 0.25% trypsin (35556-44, 5 g/L trypsin/5.3 mmol/L-EDTA, Nacalai tesque, Inc. Kyoto, Japan) at 37 °C, the cells were reconstituted in DMEM with 10% FBS and grown in fresh culture dishes. When 80% confluence was achieved, cells were passaged, and we utilized third-passage cells in all experiments attributed to their high growth rate, which was advantageous for further assessments.

### 4.3. Characterization of r-AdMSCs’ Morphology and Immunophenotypes

The morphology of cultured cells from different sites was evaluated using an optical microscope (Cell Sens Standard; Olympus; Tokyo, Japan). Flow cytometry was performed to assess the surface marker clusters of differentiation of the MSCs (CD 90), endoglin (CD 105), and hematopoietic cells (CD 34 and CD 45) [34,66]. Third-passage cultured cells were separated using trypsin and resuspended in PBS to prepare the cell suspension. Following counting, cells were incubated with the specific antibodies or corresponding isotype control antibodies presented in Table 1 at an optimal concentration of 1:500 in the dark for 30 min at 4 °C. For CD 105 and CD 34, the primary antibodies and cell preparations were subsequently treated with the secondary antibody goat anti-mouse IgG H&L (Alexa Fluor^®^ 488) (1:500, ab150113, Abcam, Cambridge, UK) in the dark for 30 min at 4 °C. The cell preparations were analyzed using a CytoFLEX flow cytometer (Beckman Coulter, Brea, CA, USA) equipped with a blue laser (488 nm), and the analysis of data was accomplished using the software CytExpert version 2.3 (Figure 7).

### 4.4. Expression of Pluripotent Genes in r-AdMSCs

The ability of the r-AdMSCs harvested from various sites to express the pluripotency genes was tested using reverse-transcriptase PCR (RT-PCR). Octamer-binding transcription factor 4 (*Oct4*), embryonic stem-cell-specific homeobox protein (*Nanog*), SRYbox-containing gene 2 (*Sox 2*), reduced expression 1 (*Rex-1*), and telomerase reverse transcriptase (*Tert*) genes were utilized. To assess the efficacy of PCR, β-actin was employed as a control (Table 2). Total RNA from r-AdMSCs of the third passage from different harvesting sites was isolated using the FastGene RNA Premium Kit (Nippon Genetics, Tokyo, Japan). The quantity and quality of RNA were assessed using a NanoDrop 2000 ultra-micro spectrophotometer (Thermo Fisher Scientific, Cambridge, MA, USA). Then, the cDNA was synthesized via the reverse transcription of 1 µg of RNA (OD_260_/OD_280_ ≈ 2.0) by applying the ReverTra Ace™ qPCR RT Master Mix kit (Toyobo Co., Ltd., Osaka, Japan). For PCR amplification, the resultant cDNA was utilized. PCR cycling was accomplished in a Veriti Thermal Cycler (Thermo Fisher Scientific, Waltham, MA, USA) according to [30]. All examinations were carried out in triplicate. To visualize the PCR products, gel electrophoresis was performed (Figure 7).

### 4.5. In Vitro Assessment of Cell Heterogeneity and Multilineage Differentiation Capacity

To assess the multilineage induction capacity of different cells from various sites, third-passage cells were induced under diverse environments to differentiate into adipogenic, osteogenic, and chondrogenic lineages. Negative control cells (1 × 10^5^ cells/well) were grown on normal media (DMED + FBS 10%) [68] (Figure 7).

#### 4.5.1. Adipogenic Differentiation

Cells were seeded at a density of 1 × 10^5^ cells in 6-well plates (for cytochemical staining and RNA isolation) and grown in standard DMEM growth medium. At 90–100% confluence, the medium was replaced in all test wells with adipogenic induction medium supplemented with 10% FBS and with adipogenic induction mixture containing 1% Pen-Strep, 1 µM dexamethasone (D4902, Sigma Aldrich, St. Louis, MI, USA), 500 µM isobutyl methyl xanthine (IBMX, I5879-100MG, Sigma Aldrich, St. Louis, MI, USA), 200 µM indomethacin (I7378-5G, Sigma Aldrich, St. Louis, MI, USA), and 5 μg/mL insulin (I5500-50MG, Sigma Aldrich, St. Louis, MI, USA). The adipogenic induction medium was used for 10 successive days and changed every 2–3 days. Experimental control cells were supplied by the normal culture media (DMEM + 10% FBS). Cells differentiated into adipocytes after 10 days were rinsed twice with PBS, and the lipid droplets accumulated in the cytoplasm were stained with oil red O stain (O1391-250ML, Sigma Aldrich, St. Louis, MI, USA) and then photographed using an inverted microscope.

#### 4.5.2. Osteogenic Differentiation

For staining and RNA extraction, 5 × 10^4^ cells were plated in 6-well plates and cultured in a typical DMEM growth medium. Osteogenic induction medium supplemented with DMEM containing 10% FBS, 1% Pen-Strep, 0.1 μM dexamethasone, 10 mM β-glycerophosphate (G9422-10G, Sigma Aldrich, St. Louis, MI, USA), and 50 mM ascorbic acid (A4544-100G, Sigma Aldrich, St. Louis, MI, USA) was added to test wells at 80% confluence. Osteo-induction was continuous for 21 days, and the regular changing of the medium was accomplished twice a week. Cells grown in ordinary DMEM medium were the experimental controls. The mineralization of the cell layer was examined using alizarin red S (ALZ, TMS-008-C, Sigma Aldrich, St. Louis, MI, USA) stain. After washing with pure water, the cell layers were examined using an inverted microscope and photographed.

#### 4.5.3. Chondrogenic Induction

Cells harvested from different sites at the third passage were cultured at a density of 5 × 10^4^ cells/well and supplied with a chondrogenic induction medium free of serum (Cat.No.C-28012, Promo Cell, GmbH, Heidelberg, Germany). The medium was changed every 2–3 days for 21 days. Alcian blue (TMS-010-C, Sigma Aldrich, St. Louis, MI, USA) staining was conducted and visualized via inverted microscopy to measure the formation of sulfated proteoglycans in the chondrogenic matrix.

#### 4.5.4. Quantitative Assessment of the Differentiation Capacity Using RT-qPCR

Using a FastGene RNA Premium Kit (Nippon Genetics, Tokyo, Japan), total RNA was extracted from the differentiated r-AdMSCs from diverse harvesting sites following the manufacturer’s protocol. Using a NanoDrop 2000 ultra-micro spectrophotometer (Thermo Fisher Scientific, Cambridge, MA, USA), RNA amount and purity (OD_260_/OD_280_ ≈ 2.0) were assessed. Then, 1 µg of RNA was utilized to synthesize the cDNA using a PrimeScript RT reagent Kit (Takara Bio, Shiga, Japan). The expression level of different multipotent genes was quantified using the StepOnePlus™ Real-Time PCR System (Thermo Fisher Scientific, Waltham, MA, USA). We utilized the primers presented in Table 3. For adipogenesis, adiponectin (AdipoQ) and complement factor D (CFD) genes were used. Meanwhile, for osteogenesis, osteopontin (OPN), bone sialoprotein (BSP), and bone morphogenetic protein 2 (BMP2) were used. For chondrogenic differentiation evaluation, aggrecan (ACAN), chondromodulin-1 (CHM1), and collagen type 2 (COL2A1) were employed. The qPCR system contained 1 µL cDNA, 0.5 µL forward primer, 0.5 µL reverse primer (10 µmol/L), 10 µL THUNDERBIRD^®^ Next SYBR^®^ qPCR Mix (Toyobo Life Science, Osaka, Japan), and 8 µL ddH_2_O. The 2^−∆∆Cq^ method was used to calculate the relative quantification [35], which was then normalized to β-actin. Data are displayed as expression levels normalized to that of the control.

### 4.6. Statistical Analysis

Comparison between different r-AdMSC harvesting sites was performed using one-way analysis of variance (ANOVA) and Tukey’s post hoc test. The statistical analyses were conducted using GraphPad Prism software version 9 (GraphPad Software, Inc., La Jolla, CA, USA). Data are expressed as mean ± standard error of the mean (SEM), and *p* < 0.05 was defined as being significant.

### 4.7. Limitations of the Present Study

In cell-based therapies, the source of the cells is ought to be selected according to the targeted application. In the current study, we could provide an insight into the impact of the fat depot site collection on the differentiation potential of the isolated AdMSCs. These findings may help to define the optimum cell source for clinical applications, including regenerative medicine or tissue engineering. However, the implementation of novel bioinformatic approaches in future experiments could be useful to elaborate upon the mechanisms behind these effects. Several pre-clinical investigations are still required before the clinical applications. The incorporation of the computational (in silico) studies enables more practical, cost-effective studies compared to in vivo approaches, which are carried out in complete organisms. Additionally, computational techniques restrict the usage of animal models in research, which is consistent with the justification for creating novel, secure therapeutic candidates.

## 5. Conclusions

The present study confirmed the crucial impact of the adipose tissue harvesting site on the differentiation capacity of isolated r-AdMSCs. The epididymal and inguinal cells were superior at the adipogenic and osteogenic lineages. However, the subcutaneous cells were superior in the chondrogenic lineage induction. This important factor should be considered during the employment of AdMSCs in cell-based applications in tissue engineering and regenerative medicine. In the future, we hope to provide strategies that enable the depot-specific manipulation of these fat pads.

## Figures and Tables

**Table 1:** Specifics of antibodies employed for flow cytometric analysis.

Antigen	Primary Antibody	Secondary Antibody	Isotype Control Antibody
**CD 90**	FITC anti-rat CD90 (206105, BioLegend, San Diego, CA, USA)	----	FITC Mouse IgG1, κ (981802, BioLegend, San Diego, CA, USA)
**CD 105**	Mouse anti-CD105 (OTI8A1) (ab156756, Abcam, Cambridge, UK)	Goat anti-mouse IgG H&L (Alexa Fluor^®^ 488) (ab150113, Abcam, Cambridge, UK)	Purified Mouse IgG2b, κ (402202, BioLegend, San Diego, CA, USA)
**CD 45**	FITC anti-rat CD45 (202205, BioLegend, San Diego, CA, USA)	----	FITC Mouse IgG1, κ (981802, BioLegend, San Diego, CA, USA)
**CD 34**	Mouse anti CD34 (ICO115) (SC-7324, Santa Cruz Biotechnology, Inc., CA, USA)	Goat anti-mouse IgG H&L (Alexa Fluor^®^ 488) (ab150113, Abcam, Cambridge, UK)	Purified Mouse IgG1, κ (401402, BioLegend, San Diego, CA, USA)

**Table 2:** Specific primers used for RT-PCR for pluripotent genes.

Name	Direction	Primer Sequence (5′-3′)	Accession Number
***Oct 4***	F	CGAACCTGGCTAAGCTTCCA	NM_001009178.2
	R	GCCATCCCTCCACAGAACTC	
***Nanog***	F	TACCTCAGCCTCCAGCAGAT	XM_006237310.3
	R	CATTGGTTTTTCTGCCACCT	
***Sox 2***	F	CTCGCAGACCTACATGAAC	NM_001109181.1
	R	TCGGACTTGACCACAGAG	
***Rex-1***	F	GCTCCGGCGGAATCGAGTGG	XM_032907726.1
	R	GCACGTGTTGCTTGGCGACC	
***Tert***	F	CCCGAGTATGGCTGCATGAT	NM_053423.1
	R	AAAGTCCGAGTGTCCAGCAG	
**β-actin**	F	GCAGGAGTACGATGAGTCCG	[67]
	R	ACGCAG CTCAGTAACAGTCC	

**Table 3:** Gene primers used for RT-qPCR to quantitatively assess the differentiation power of various r-AdMSCs from different sites.

Purpose	Name	Direction	Primer Sequence (5′-3′)	Refs.
**Adipogenic induction**	**ADIPOQ**	F	TAATTCAGAGCAGCCCGTAG	[69]
		R	TGGGGATAACACTCAGAACC	
	**CFD**	F	GGAGTGACCAAGGATGAGG	[69]
		R	ACCCAGTGAGGCATTGTG	
**Osteogenic induction**	**BMP2**	F	CAGGTCTTTGCACCAAGATG	[70]
		R	GCTGGACTTAAGACGCTTCC	
	**OPN**	F	GAAGAGCCAGGAGTCCGATG	[30]
		R	CTTCCCGTTGCTGTCCTGAT	
	**BSP**	F	AGGCTACGAGGGTCAGGATT	[30]
		R	CTCTGCCTCCCGTGAAAC	
**Chondrogenic induction**	**ACAN**	F	CTCTGCCTCCCGTGAAAC	[71]
		R	TGAAGTGCCTGCATCTATGT	
	**COL2A1**	F	TCCTAAGGGTGCCAATGGTGA	[72]
		R	AGGACCAACTTTGCCTTGAGGAC	
	**CHM1**	F	GAGAACTGTGAGGGCTGTCA	[30]
		R	GATACCTCGGGCCAGAAGTG	
**Internal control**	**β-actin**	F	GCAGGAGTACGATGAGTCCG	[67]
		R	ACGCAGCTCAGTAACAGTCC	

## Data Availability

The original contributions exhibited in the study are included in the article; further inquiries can be directed to the corresponding author/s.
